# Supplementary material for: Single-Cell Analysis Reveals that Chronic Silver Nanoparticle Exposure Induces Cell Division Defects in Human Epithelial Cells
Source: Int J Environ Res Public Health. 2019 Jun 11;16(11):2061. doi: 10.3390/ijerph16112061 (PMC6603987; doi:10.3390/ijerph16112061)
Supplement: Supplementary file 1 [file ijerph-16-02061-s001.zip › Garcia et al_2018_IJEPH_SupplMat_R1.pdf]

Single-cell analysis reveals that chronic silver nanoparticle exposure induces cell division defects in human epithelial cells

Ellen B. Garcia[1], Cynthia Alms[1,4], Albert W. Hinman[1,5], Conor Kelly[1], Adam Smith[1], Marina Vance[2,6], Jadranka Loncarek[3], Linsey C. Marr[2], and Daniela Cimini[1]*

[1]Department of Biological Sciences and Fralin Life Sciences Institute, Virginia Tech, Blacksburg, VA 24061, USA
[2]Department of Civil and Environmental Engineering, Virginia Tech, Blacksburg, VA 24061, USA
[3]Center for Cancer Research, National Institute of Health, Frederick, MD 21702, USA
[4]Current affiliation: Lake Erie College of Osteopathic Medicine (LECOM) at Seton Hill, Greensburg, PA 15601, USA
[5]Current affiliation: Department of Genetics, Stanford University School of Medicine, Stanford, CA 94305, USA
[6]Current affiliation: Department of Mechanical Engineering, University of Colorado Boulder, Boulder, CO 80309, USA

* Correspondence: cimini@vt.edu

Supplemental Figures

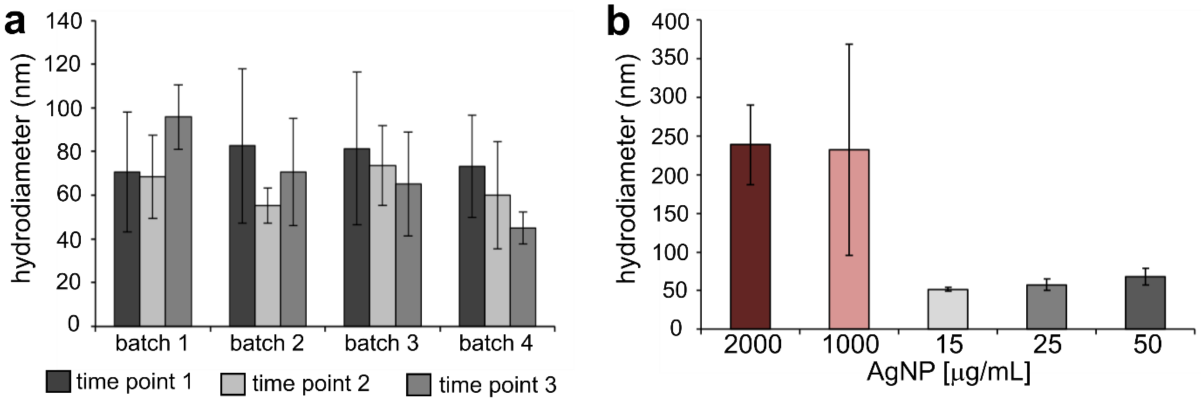

Sample	Average PDI	Average Zeta
batch 1	0.40	N/A
batch 2	0.59	N/A
batch 3	0.44	N/A
batch 4	0.42	-0.17

Sample	Average PDI	Average Zeta
2000 μg/ml	0.37	-0.06
1000 μg/ml	0.38	-0.22
15 μg/ml	0.52	-0.12
25 μg/ml	0.55	-0.07
50 μg/ml	0.53	-0.25

Figure S1. Dynamics light scattering measurements of AgNPs suspended in water (**a**) or tissue culture medium (**b**).

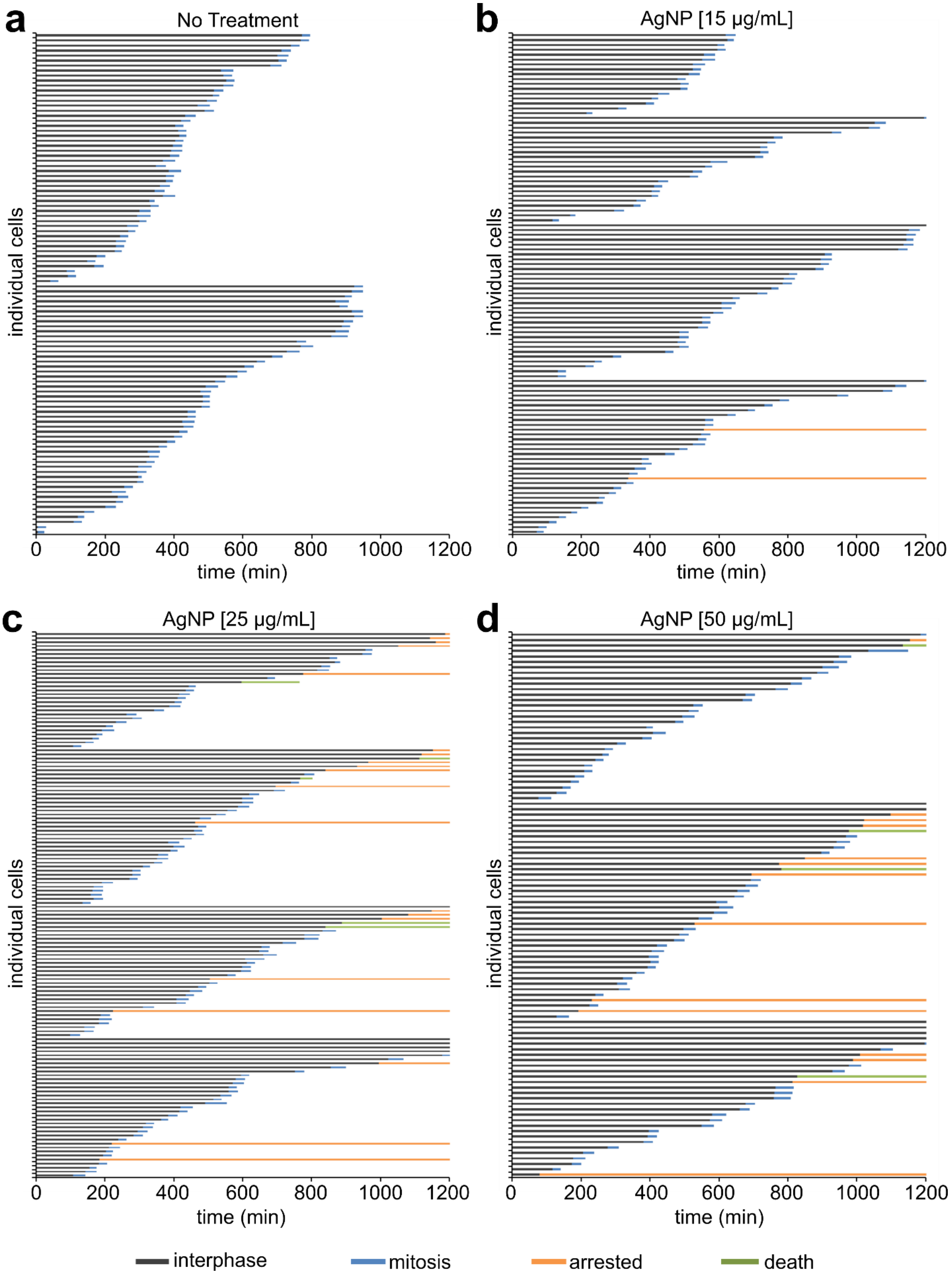
a
No Treatment
individual cells
0
200
400
600
800
1000
1200
time (min)
b
AgNP [15 μg/mL]
c
AgNP [25 μg/mL]
d
AgNP [50 μg/mL]
interphase
mitosis
arrested
death

Figure S2. Single-cell phenotypes from live cell imaging. All cells were imaged over a 20-hour period using phase contrast microscopy. Each bar represents an individual cell and the black portion of the bar represents the time each individual cell spent in interphase. The blue portion represents the time spent in mitosis by cells that progressed through a normal mitotic cell division. The orange portion represents the time cells spent in an arrested state after rounding up. The green portion refers to cells that displayed signs of cell death and represents the time elapsed between rounding up and cell death. Each graph reports subsets of cells from either a control population **(a)** or populations treated with various doses of AgNPs **(b-d)**, as indicated.

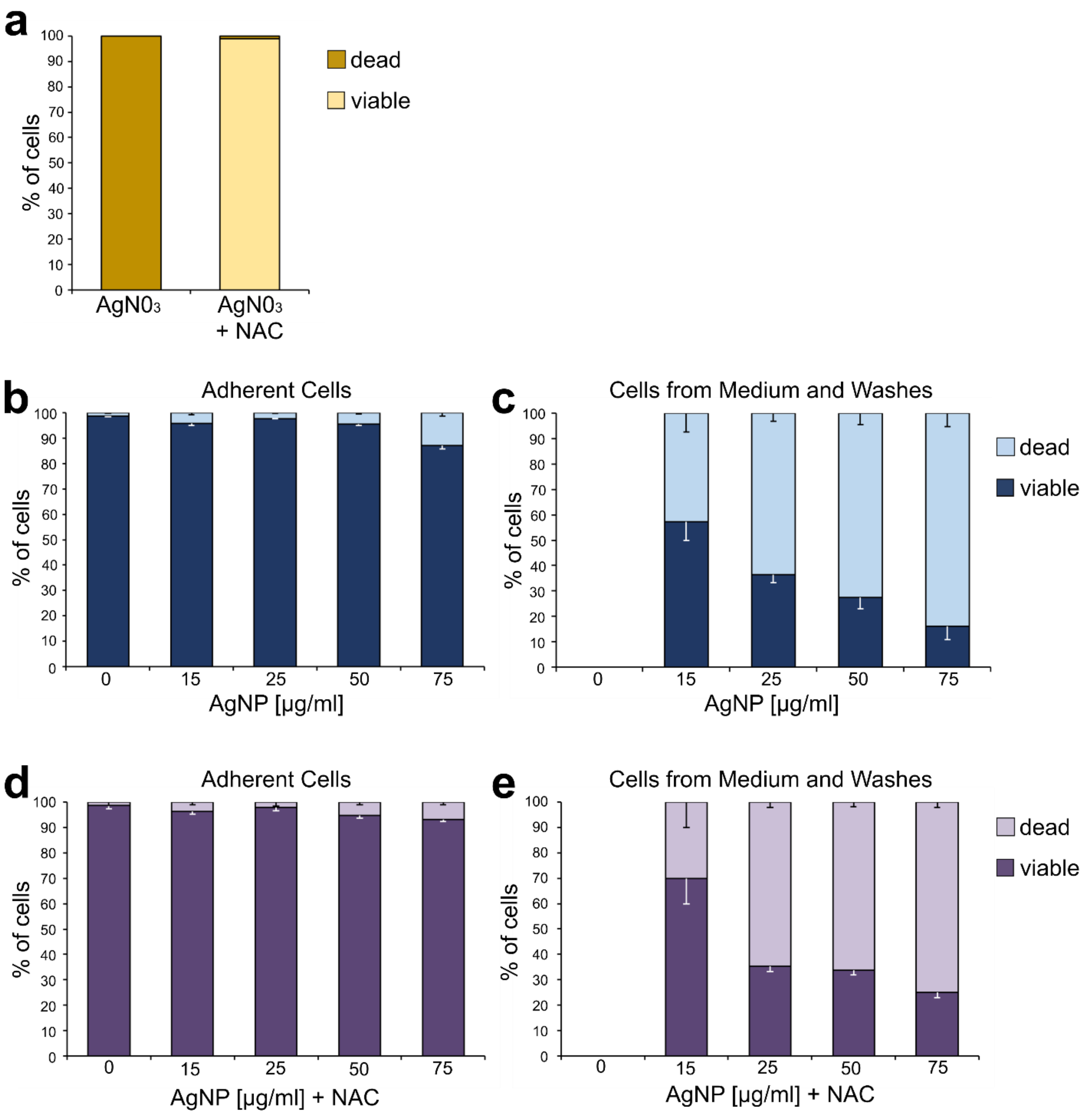

Figure S3. Cell viability assay after silver treatment. (**a**) Cells were treated with a silver ion source (50 µg/ml $AgNO_3$) with or without a silver ion chelator (NAC) for 24 hours and cell viability was quantified using trypan blue staining. (**b-c**) Cells were treated with various doses of AgNPs and cell viability was assessed in adherent (**b**) and non-adherent (**c**) cells. (**d-e**) Cells were treated with various doses of AgNPs in combination with the silver ion chelator NAC for 24 hours and cell viability was assessed in adherent (**d**) and non-adherent (**e**) cells.

Video Legends

Video S1. Representative video showing untreated RPE-1 cells (corresponding to Fig. 2a, untreated).

Video S2. Representative video showing RPE-1 cells engulfing AgNPs present in the extracellular space (corresponding to Fig. 2b).

Video S3. Representative video showing a binucleate cell undergoing tripolar cell division (corresponding to Fig. 8c).
